# Artificial Intelligence as an Enabler of Achieving Primary Care + Public Health = 1

**DOI:** 10.3389/ijph.2022.1605257

**Published:** 2022-10-12

**Authors:** Jordan Adams

**Affiliations:** Unit for Health Promotion Research, Department of Public Health, Faculty of Health Sciences, University of Southern Denmark, Esbjerg, Denmark

**Keywords:** public health, COVID-19, machine learning, global health, primary care, artificial intelligence

## Introduction

Following the turn of the century, Artificial Intelligence (AI) research has increased exponentially, resulting in a vast number of novel applications. Collectively, these are becoming an integral entity in the optimisation of public health and primary care. This commentary article is based upon a scoping review, which attempts to provide an overview of these relevant AI-applications - necessitated by AIs rapidly proliferating nature.

The AI developments seen in the sector of public health and primary care are largely owed to advances in computational power and data access, which combined, bolster the predictive power of Machine Learning algorithms. The profound ability of these algorithms to recognise associations too subtle or niche for human intellect has endorsed their presence across the entire spectrum of medicine and health. Within public health, this predominantly relates to screening, disease surveillance, prediction and environmental decision making, but through an extended scope of public health, includes the prevention of crime and inner-city pollution alongside the optimisation of transport infrastructures, education systems and health services. Likewise, in the case of primary care, machine and deep learning are proving invaluable to the process of diagnosis, as their self-improving nature helps negate current diagnostic and classification errors. Concerning treatment, AI can assist in drug discovery for rare genetic diseases, optimise vaccine development and formulation, and with assistance from multi-omics and Big Data, foster a transition towards personalised medicine.

The extent to which these aforementioned applications manifest beneficially is currently ambiguous and somewhat undetermined. Therefore, a literature search was carried out to define the extent by which AI collectively augments primary care and public health. Public Health was defined as “the art and science of preventing disease, prolonging life and promoting health through the organized efforts of society” [1]. The sub-domains discoursed within the final 372 studies were increasingly diverse, including topics such as the environment, policy making, ethics, quality of life and sub-themes of healthcare.

These sub-themes were subsumed under the umbrella term of public health, partly due to the fact that many of the multi-themed articles, e.g., reviews, failed to distinguish between care and public health applications. In presenting them as inextricable entities, this highlights the degree to which AI in both primary care and public health conjoin to form a complete and optimal health approach or namely, that primary care + public health = 1. It also posits that to some degree, this is already actualised, with the ubiquity of AI growing and uniting humanities approach in both care and health domains. Several justifications for this are discussed below.

## Why in the Case of AI, Primary Care + Public Health = 1

AIs profound abilities to analyse subtle trends within expansive data sets [such as those from Big Data sources such as Omics, Patient records, EMRs, social media, Internet of Things (IoTs)], is revolutionary in both public health and health care. In health care this fosters the uprising of Precision Medicine and Evidence-based decision making, through the following process—which can be equally effective translated into public health settings. The ongoing monitoring and real-time analysis of each patient is represented in digitalised patient records and EMR’s, and as stated can be supplemented by other Big Data sources. Collective AI analysis of this data on a populational scale can then determine optimal treatment programs accounting for individualistic differences. This precision medicine can transcend the frame of primary care, and evolve into Precision Public Health (PPH), defined as “providing the right intervention to the right population at the right time” [2]. Going forward, this area of public health has huge potential to reduce the health disparities within populations by using the best available data to target those most in need. This same aptitude is highly relevant to AI in environmental health, predicting natural disasters, optimising responses and informing prospective policies and strategies.

The pending widespread assimilation of AI in administrative and data-handling roles also highlights the link between the AI in primary care and public health. In reducing the workload and occupational burden of health professionals, doctors are permitted to delegate more time to fewer patients in empathetic and supportive care roles. Assistance from novel health technologies and the Internet of Things (IoTs) will further relieve the burden on primary care practitioners by favouring prevention and self-help methods.

## Lessons From COVID 19

Using a narrower scope, the entanglement and co-dependency of public health and healthcare is exemplified *via* AIs involvement in the COVID-19 pandemic. AI was utilised on every defensive front to combat the virus. Some articles explicated narrow uses like drug repurposing and diagnosis procedures, whilst others applied a broader public health definition encompassing policy making, surveillance and health education.

Regarding healthcare, Sarker et al. [3] investigated the use of AI and robotics within “In-Patient Care” which encompasses the primary care domains of Diagnosis, Evaluation, Treatment. A deep learning phone application was able to diagnose and categorise COVID-19 cases *via* CT Scans, X-rays and breathing recordings on phone application [4]- a technology that shows potential for pneumonia and major non-communicable diseases such as asthma. Post-diagnosis, AI can continue to predict the risk of developing critical illnesses, permitting adjustments to subsequent in-hospital allocations and courses of care. Linking this to public health, this information is subsequently subsumed into a collective knowledgebase from which AI can monitor and analyse. The results can be used to inform policy decisions and recommendations for ‘at risk groups’ which disseminated *via* AI based chatbots, highlighting a partnership between AI on a care and populational level. Some studies extended the scope by explicating the role of chatbots in addressing the mental health issues that manifested as a biproduct of the pandemic [5]. In this sense, newfound knowledge and AI applications that arose from the pandemic can be applied to address alternative health concerns, especially in the areas of surveillance, drug-repurposing and decision making (both clinical and potentially in the near future, political and environmental).

### Conclusion

Overall, AI is becoming an integral entity of both primary care and public health and as such, will prove an unprecedented facilitator in optimizing global health approaches. The pandemic exemplifies the importance of a unified, multi-level approach whereby AI is deployed on both a personal and populational front. These differing channels of implementation are somewhat co-dependant and collaboration between the two will prove essential in resolving the impending threat from climate change, non-communicable diseases and further epidemics. Furthermore, AIs expediency within settings involving limited resources or medical expertise provide a valuable opportunity to combat global poverty and health inequalities.

The continuation of AIs exponential growth necessitates a public health response involving collaborative and long-term policies and strategies that prioritise ethical standards and the further amalgamation of efforts in both primary care and public health. Afterall is it the coalesced efficacy of these sub domains that determine the extent to which a health approach is deemed optimal.
